# *FIP1* Plays an Important Role in Nitrate Signaling and Regulates *CIPK8* and *CIPK23* Expression in *Arabidopsis*

**DOI:** 10.3389/fpls.2018.00593

**Published:** 2018-05-04

**Authors:** Chao Wang, Wenjing Zhang, Zehui Li, Zhen Li, Yingjun Bi, Nigel M. Crawford, Yong Wang

**Affiliations:** ^1^National Key Laboratory of Crop Biology, College of Life Sciences, Shandong Agricultural University, Tai’an, China; ^2^Section of General Biology, Department of Life Science and Engineering, Jining University, Jining, China; ^3^Section of Cell and Developmental Biology, Division of Biological Science, University of California at San Diego, La Jolla, CA, United States

**Keywords:** *Arabidopsis*, *FIP1*, *CPSF30-L*, nitrate signaling, nitrate uptake and assimilation, *CIPK8*, *CIPK23*

## Abstract

Unraveling the molecular mechanisms of nitrate regulation and deciphering the underlying genetic network is vital for elucidating nitrate uptake and utilization in plants. Such knowledge could lead to the improvement of nitrogen-use efficiency in agriculture. Here, we report that the *FIP1* gene (factor interacting with poly(A) polymerase 1) plays an important role in nitrate signaling in *Arabidopsis thaliana. FIP1* encodes a putative core component of the polyadenylation factor complex. We found that FIP1 interacts with the cleavage and polyadenylation specificity factor 30-L (CPSF30-L), which is also an essential player in nitrate signaling. The induction of nitrate-responsive genes following nitrate treatment was inhibited in the *fip1* mutant. The nitrate content was also reduced in *fip1* seedlings due to their decreased nitrate uptake activity. Furthermore, the nitrate content was higher in the roots but lower in the roots of *fip1*, which may result from the downregulation of *NRT1.8* and the upregulation of the nitrate assimilation genes. In addition, qPCR analyses revealed that FIP1 negatively regulated the expression of *CIPK8* and *CIPK23*, two protein kinases involved in nitrate signaling. In the *fip1* mutant, the increased expression of *CIPK23* may affect nitrate uptake, resulting in its lower nitrate content. Genetic and molecular evidence suggests that *FIP1* and *CPSF30-L* function in the same nitrate-signaling pathway, with FIP1 mediating signaling through its interaction with CPSF30-L and its regulation of *CIPK8* and *CIPK23*. Analysis of the 3′-UTR of *NRT1.1* showed that the pattern of polyadenylation sites was altered in the *fip1* mutant. These findings add a novel component to the nitrate regulation network and enhance our understanding of the underlying mechanisms for nitrate signaling.

## Introduction

Nitrogen is an essential macronutrient, and its availability in soil is a major limiting factor for plant growth and development. N fertilizers are routinely used to increase agricultural productivity; however, the low NUE of many crops means that a large portion of the N application cannot be absorbed by plants and is lost to the environment, leading to various environmental and ecological problems, such as eutrophication and soil acidification, as well as increased economic costs for farmers (Canfield et al., 2010). Improving NUE and understanding how plants regulate their growth and development in response to different levels and forms of N available in the rhizosphere are essential for addressing these problems and improving the sustainability of agriculture.

Nitrate is the main source of inorganic N for terrestrial plants, which have evolved sophisticated regulatory mechanisms to withstand changing nitrate concentrations in the environment (Crawford and Glass, 1998). In the *Arabidopsis thaliana* genome, four gene families (*NRT1/PTR*, *NRT2*, *CLC*, and *SLAC1*/*SLAH*) are responsible for nitrate absorption and distribution (Krapp et al., 2014). Once absorbed into the cells, a portion of the nitrate is reduced to nitrite and then to ammonium by the action of NR and NiR, respectively (Crawford and Glass, 1998). The ammonium is then assimilated into glutamine by glutamine synthetase (Stitt, 1999).

Nitrate serves not only as a nutrient, but also as a potent signal regulating the long-term and short-term development and physiology of plants. In the long term, nitrate affects the metabolism, growth, and development of *Arabidopsis* (Wang et al., 2007; Gutiérrez, 2012; Vidal et al., 2013). Several essential genes have been found to be involved in regulating the effects of nitrate on root architecture, including *ANR1*, *NRT1.1* (*NITRATE TRANSPORTER 1.1*, also called *NPF6.3* and *CHL1*), *AFB3-miR393*, *NLP7*, *TCP20*, *HRS1*, and *HHO1* (Zhang and Forde, 1998; Castaings et al., 2009; Ho et al., 2009; Wang et al., 2009; Vidal et al., 2010, 2013; Guan et al., 2014, 2017; Medici et al., 2015). Oligopeptide signals have also been shown to mediate N-dependent root architecture via the CLE-CLAVATA1 module (Araya et al., 2014) and via leucine-rich repeat receptor kinases (LRR-RKs) (Tabata et al., 2014).

In the short term, nitrate induces the PNR in roots and shoots, during which the expression of more than 1000 genes can be rapidly altered (Wang et al., 2003, 2007; Scheible et al., 2004; Krouk et al., 2010a; Alvarez et al., 2012). Important nitrate regulatory genes have been identified over the last decade that play crucial roles in regulating nitrate-responsive genes such as *NRT1.1*, *NRT2.1*, *NRT2.2*, *NITRATE REDUCTASE 1* (*NIA1*), *NIA2*, and *NiR* (Castaings et al., 2009; Alvarez et al., 2014; Guan et al., 2014, 2017; Xu et al., 2016; Li et al., 2017). The only nitrate sensor to have been identified thus far, *NRT1.1*, triggers nitrate responses, regulating the expression of *CIPK8* as a positive factor and *CIPK23* as a negative factor during PNR (Ho et al., 2009; Hu et al., 2009). The CIPK23-CBL9 protein complex has been implicated in the switch between the dual affinities of NRT1.1, through the phosphorylation of a threonine residue (Thr101) (Ho et al., 2009). *NLP6* and *NLP7* act as key activators of nitrate assimilatory genes (Castaings et al., 2009; Konishi and Yanagisawa, 2013; Marchive et al., 2013). *NRG2*, another recently identified regulator, was found to act upstream of *NRT1.1* and interact with NLP7 in the nucleus (Xu et al., 2016). *LBD37/38/39* function as negative regulators that repress the expression of a subset of genes involved in nitrate uptake and assimilation (Rubin et al., 2009). TARGET (transient assay reporting genome-wide effects of transcription factors) and ChIP-sequencing analyses were used to identify roles for *SPL9* and *bZIP1*, respectively, in the PNR (Krouk et al., 2010b; Para et al., 2014; Vidal et al., 2015). Both *TGA1* and *TGA4* are induced by nitrate treatments and involved in nitrate transport and metabolic functions (Alvarez et al., 2014; O’Brien et al., 2016). Furthermore, TGA1 can interact with CIPK23, suggesting that phosphorylation may be important for TGA1 activation (Yazaki et al., 2016).

Recently, a 65-kDa subunit of the CPSF *CPSF30-L* was found to function upstream of *NRT1.1* in nitrate signaling, where it affects nitrate uptake and assimilation (Li et al., 2017); however, the structure of the regulatory network modules and the underlying molecular mechanisms remain uncharacterized. *CPSF30* has two spliced forms, a larger one (*CPSF30-L*) and a smaller one (*CPSF30-S*) (Delaney et al., 2006). CPSF30-S interacts with FIP1 (factor interacting with poly(A) polymerase 1), an important regulator of the nuclease activity of CPSF30 (Addepalli and Hunt, 2007); however, whether CPSF30-L can interact with FIP1 has yet to be reported. Here, we demonstrate that FIP1 interacts with CPSF30-L and plays an important role in nitrate signaling. Our results show that *FIP1* also modulates the nitrate content in plants by regulating their nitrate transport, allocation, and assimilation. Moreover, *FIP1* negatively regulates the expression of *CIPK8* and *CIPK23*. Molecular and genetic analyses revealed that *FIP1* and *CPSF30-L* function in the same nitrate signaling pathway.

## Materials and Methods

### Plant Materials

*Arabidopsis thaliana* (Columbia-0 ecotype) and homozygous transgenic seeds containing the NRP-YFP construct (SS204-9) (Wang et al., 2009) were used as the wild types (WTs). The mutant lines *chl1-13* (original name: Mut21; containing a NRT-YFP construct) (Wang et al., 2009), *cipk8-1* (Hu et al., 2009), *cipk23-3* (Ho et al., 2009), *nrg2-3* and *nlp7-4* (Xu et al., 2016), and *cpsf30* (original name: Mut65; containing a NRT-YFP construct) (Li et al., 2017) were described previously. The *fip1* mutant (Salk_087117), containing a T-DNA insertion in the sixth exon of *FIP1*, was obtained from ABRC and used for further analysis (Alonso et al., 2003). The construct p35S::*FIP1* in destination vector pMDC43 (Thermo Fisher Scientific) was transformed into the *fip1* mutant using the *Agrobacterium*-mediated floral dip method (Xu et al., 2016). Homozygous transgenic lines were isolated as complementation line (*FIP1*/*fip1*) for further investigation.

### Growth and Treatment Conditions

Seeds were germinated on nylon mesh floating in a 2.5-mM ammonium succinate [(NH4)_2_Suc] solution for 7 days. To detect the expression of the nitrate-responsive genes, the roots were treated with 10 mM KNO_3_ or KCl for 2 h and then harvested. For fluorescence microscopy, seedlings were grown on the KNO_3_ medium for 4 days before being observed using a Nikon Eclipse Ti-S microscope (Nikon, Tokyo, Japan). The fluorescence intensity was quantified using Image J (Schneider et al., 2012).

To determine expression profiles, various tissues were harvested either from plants grown for 7 days in ½ MS medium (pH = 5.7, 10 mM KNO_3_, and 10 mM NH_4_NO_3_) or for 6 weeks in soil. Roots and shoots were harvested from seedlings grown in ½ MS solution for 7 days and used to determine their nitrate concentration, NR activity, and amino acid content. The expression levels of the genes involved in nitrate transportation and assimilation were determined in the roots and shoots of these seedlings, whereas the expression of the nitrate regulatory genes was measured in seedlings grown in 2.5 mM (NH_4_)_2_Suc, 10 mM KNO_3_, and 10 mM NH_4_NO_3_ for 7 days.

### qPCR Analysis

Total RNA was isolated from *Arabidopsis* roots and shoots using a Total RNA Miniprep Kit (CWBIO, Beijing, China). cDNA synthesis was carried out using the RevertAid first-stand Synthesis System Kit (Thermo Fisher Scientific, Waltham, MA, United States). An UltraSYBR Green Mixture qPCR Kit (CWBIO) was used for the qPCR reaction, following the manufacturer’s protocol. Gene expression was determined by real-time PCR using an ABI7500 Fast Real-Time PCR System (Thermo Fisher Scientific). *TUB2* (At5g62690) was used as the internal reference gene.

### Expression Profile Analysis

A GUS assay was performed according to Xu et al. (2016). A 381-bp genomic sequence located upstream of the *FIP1* start codon was cloned into the pMDC163 destination vector (Thermo Fisher Scientific). Transgenic plants containing the *Pro_*FIP1*_*::*GUS* construct were grown on ½ MS for 7 days or in soil for 6 weeks before their GUS activity was assessed.

### Nitrate, NR Activity, and Amino Acid Content Assays

Plant nitrate content was determined using the salicylic acid method, as described previously (Zhao and Wang, 2017). The amino acid content and NR activity of the seedlings were tested using a Micro Amino Acid Content Assay Kit and a Micro Nitrate Reductase (NR) Assay Kit (Solarbio, Beijing, China), respectively.

### Yeast Two-Hybrid Assays

Full-length cDNA fragments of *CPSF30-L* or *CPSF30-L* containing a point mutation in nucleotide 376 (G to A; m*CPSF30-L*) were introduced into the pGBKT7 vector (Clontech Laboratories, Mountain View, CA, United States), while a full-length cDNA fragment of *FIP1*′ a 1335-bp sequence encoding the N-terminal of *FIP1* (*FIP1*-S1), a 2196-bp sequence encoding the C-terminal of *FIP1* (*FIP1*-S2), were ligated into the pGADT7 vector (Clontech Laboratories). The two-hybrid interaction was performed following the instructions provided by the manufacturer (Clontech Laboratories).

### GST Pull-Down Assays

Full-length cDNA of *CPSF30-L* or m*CPSF30-L* was cloned into pGEX4T-1 (GE) to produce a GST-CPSF30-L or GST-mCPSF30-L product as bait protein, respectively. *FIP1* or *FIP1*-S1 was cloned into pET28a (Novagen) to produce His-FIP1 or His-FIP1-S1 product as prey protein, respectively. The constructs were introduced into *Escherichia coli* strain BL21. The preparation and immobilization of the bait protein, the preparation and capture of the prey protein, and bait-prey elution were performed using a GST protein interaction pull-down kit (Thermo). The prepared eluent was loaded into wells and electrophoresis was run in the stacking and separating gel, respectively. Following SDS-PAGE, the proteins were transferred onto blotting membrane and blocked, then adding anti-HIS mAb (Zoonbio) as primary antibody and HRP-conjugated secondary antibody (Zoonbio). The Chemiluminescent (ECL) was used to visualize protein bands as recommended by the manufacturer (Thermo).

### BiFC Analysis

Transient bimolecular fluorescence complementation (BiFC) assays in *Arabidopsis* mesophyll protoplast were performed as described (Xu et al., 2016). Full-length cDNA of *CPSF30-L* and m*CPSF30-L* were cloned into the Gateway compatible binary vectors pSITE-NEYFP vectors containing the N-terminal fragments of YFP (YFP^N^). Full-length cDNA of *FIP1* and *FIP1*-S1 were cloned into pSITE-CEYFP containing the C-terminal fragments of YFP (YFP^C^), respectively. These vectors CPSF30-L-YFP^N^ and FIP1-YFP^C^, CPSF30-L-YFP^N^ and FIP1-S1-YFP^C^, and mCPSF30-L-YFP^N^ and FIP1-YFP^C^ were cotransfected into protoplast and the empty vectors YFP^N^ and YFP^C^ were used as negative controls. The fluorescence of transfected protoplast was observed using confocal microscope (Leica TCS SP5II).

### Analysis of Polyadenylation in *NRT1.1* 3′-UTR

For analysis of *NRT1.1* 3′-UTR, the nested PCR technique was used as described (Li et al., 2017). The seedlings of WT, *fip1*, *cpsf30*, and *fip1cpsf30* were grown on 10 mM KNO_3_ for 7 days and total RNA were extracted, and then reverse transcription polymerase chain reaction (RT-PCR) was performed. The 3′-UTR of *NRT1.1* after two rounds of PCR was analyzed by polyacrylamide gel electrophoresis (PAGE).

## Results

### The Interaction Between FIP1 and CPSF30-L

**FIGURE 1:**
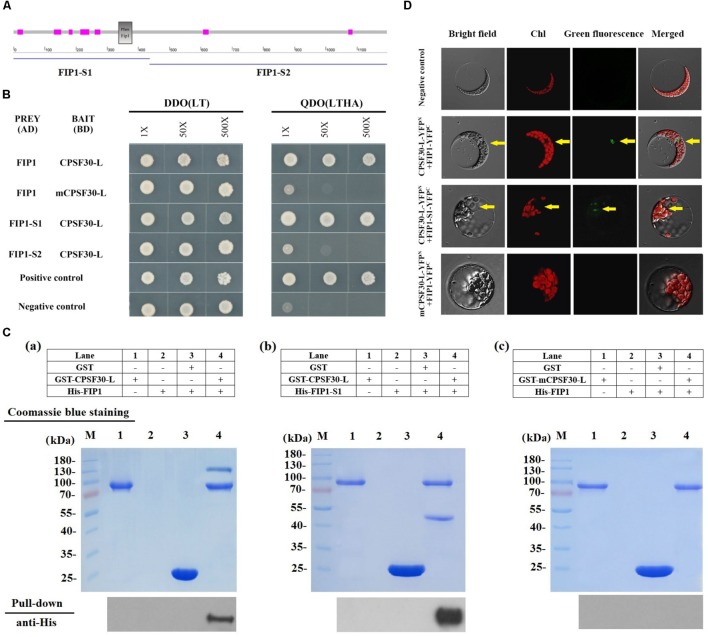
FIP1 interacts with CPSF30-L. **(A)** Secondary structure of FIP1 defined using SMART software. FIP1-S1, N-terminal of FIP1, comprising 445 amino acids with a Fip1 conserved domain (indicated by the gray box); FIP1-S2, C-terminal of FIP1, comprising 731 amino acids. The seven fuchsia boxes indicate regions of low complexity. **(B)** Yeast two-hybrid (Y2H) assay of the FIP1 and CPSF30-L interaction. CPSF30-L, a BD vector containing cDNA encoding CPSF30-L (a 65-kDa transcript of At1g30460.2); mCPSF30-L, a BD vector containing *CPSF30-L* cDNA with a point mutation at nucleotide 376 (G to A); FIP1, an AD vector containing *FIP1* cDNA (a transcript of At5g58040); FIP1-S1, an AD vector containing cDNA comprising a 1335-bp sequence encoding the N-terminal of FIP1; FIP1-S2, an AD vector containing cDNA comprising a 2196-bp sequence encoding the C-terminal of FIP1; DDO (LT), SD medium lacking leucine and tryptophan used to verify the co-transformation of AD and BD plasmids; QDO (LTHA), SD medium lacking leucine, tryptophan, histidine, and adenine used to test for autoactivation or interaction. Positive controls (BD-pGBKT7-53 and AD-pGADT7-T) and negative controls (BD-pGBKT7-Lam and AD-pGADT7-T) are also shown. **(C)** GST pull-down assay to detect the *in vitro* interaction of FIP1 and CPSF30-L. The tagged protein GST-CPSF30-L, GST-mCPSF30-L, His-FIP1, and His-FIP1-S1 were synthesized in *Escherichia coli*. His-FIP1 (a) or His-FIP1-S1 (b) was pulled down using glutathione agarose beads with GST-CPSF30-L, (c) His-FIP1 was not pulled down with GST-mCPSF30-L, as detected using an anti-His antibody. **(D)** BiFC analysis for interaction between FIP1 and CPSF30-L. CPSF30-L and mCPSF30-L were fused to N-terminal fragments of YFP, FIP1 and FIP1-S1 were fused to C-terminal fragments of YFP, respectively. Different combinations of expression vectors and negative control (indicated on the left of the panel) were transfected into *Arabidopsis* mesophyll protoplast. Presence of YFP signal indicates reconstitution of YFP through protein interaction of the tested pairs. Yellow arrows indicate the nucleus.

We first tested if FIP1 can interact with CPSF30-L using a yeast two-hybrid assay. Full length *FIP1* and *FIP1* segments were used for the assays: segment 1 comprised the N-terminal 445 amino acid residues while segment 2 contained the C-terminal 731 amino acid residues (**Figure 1A**). The CPSF30-L protein and a CPSF30-L variant containing a point mutation resulting in a conversion of Gly to Arg at the 126^th^ amino acid in the third zinc finger, mCPSF30 (Li et al., 2017), were used as bait proteins. We found that CPSF30-L and FIP1 interacted with each other; however, mCPSF30 did not interact with FIP1 (**Figure 1B**). Specifically, CPSF30-L interacted with the N-terminus but not the C-terminus of FIP1.

To confirm the interaction between FIP1 and CPSF30-L, *in vitro* GST pull-down assay and *in vivo* test with BiFC assays in *Arabidopsis* mesophyll protoplast were performed. In pull-down assay, the interaction of GST tagged CPSF30-L and His-tagged FIP1 was inspected. His-FIP1 or His-FIP1-S1 was readily pulled down by glutathione agarose beads with GST-CPSF30-L, as detected using an anti-His antibody (**Figure 1Ca,b**). When mCPSF30-L was used, His-FIP1 could not be pulled down (**Figure 1Cc**). In BiFC assay, a direct interaction was observed between FIP1 and CPSF30-L as well as FIP1-S1 and CPSF30-L in the nucleus of the protoplast when CPSF30-L-YFP^N^ was coexpressed with FIP1-YFP^C^ and FIP1-S1-YFP^C^. But no interaction was found when mCPSF30-L-YFP^N^ and FIP1-YFP^C^ were coexpressed (**Figure 1D**). These results suggest that CPSF30-L interacts with the N-terminus of FIP1, and this interaction depends on a Gly at the 126th amino acid of CPSF30-L.

### The *fip1* Mutant Is Defective in the PNR

**FIGURE 2:**
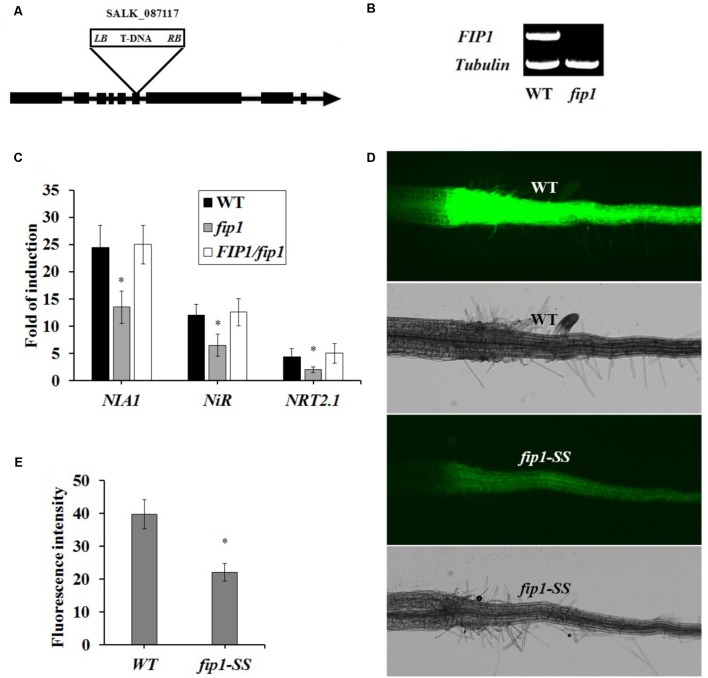
FIP1 regulates the expression of nitrate-responsive genes. **(A)** Schematic map of the T-DNA insertion site in the *fip1* mutant. Exons and introns are represented by black boxes and lines, respectively. The location of the T-DNA insertion in *FIP1* is indicated by a triangle. **(B)** RT-PCR analysis of *FIP1* mRNA levels in the wild type (WT) and *fip1*. Total RNA was isolated from 7-days-old seedlings grown on ½ MS. *TUB2* serves as the internal control. **(C)** The expression of nitrate-responsive genes in the roots of the *fip1* mutant and complementation line (*FIP1*/*fip1*). Seedlings were grown on medium with 2.5 mM ammonium succinate as the sole nitrogen source for 7 days, and then treated with 10 mM KNO_3_ or KCl as a control for 2 h. The transcripts of the nitrate-responsive genes were quantified using qPCR. Error bars represent the SD of the biological replicates (*n* = 4). Asterisks indicate significant differences (*p* < 0.05, *U*-test). **(D)** Nitrate responsiveness in 4-days-old *fip1-SS* seedlings grown on a medium containing KNO_3_, revealed using a fluorescent reporter system (NRP-YFP). **(E)** Quantification of root fluorescence in the WT and the *fip*1-*SS* mutant described in **(D)**. Error bars represent the SD of the biological replicates (*n* = 60), asterisks indicate significant differences to WT (*p* < 0.05, *U*-test).

To explore the function of *FIP1*, we obtained a *fip1* mutant (Salk_087117), containing a T-DNA insertion in the sixth exon (**Figure 2A**), from ABRC. *FIP1* expression was undetectable in this mutant (**Figure 2B**). To determine whether FIP1 is involved in the PNR, we determined the expression levels of three known nitrate-responsive genes, *NRT2.1* (encoding a high-affinity nitrate transporter), *NIA1*, and *NiR*, using qPCR. As shown in **Figure 2C**, the induction of these three genes by nitrate treatment was significantly decreased in the *fip1* mutant, but was restored to WT levels in the *FIP1/fip1* complementation line, indicating that *FIP1* plays an important role in nitrate signaling. In addition, we previously constructed a transgenic *Arabidopsis* line containing a nitrate-responsive reporter (SS204-9) that exhibited strong YFP fluorescence in the roots of *Arabidopsis* plants in the presence of nitrate (Wang et al., 2009). We introduced the nitrate-responsive reporter into the *fip1* mutant by crossing it with SS204-9 to generate *fip-SS*. The *fip1*-YFP line had a significantly reduced fluorescence in the presence of nitrate in comparison with the WT (**Figures 2D,E**). These results show that FIP1 functions as an important regulatory gene in the PNR.

To test whether *FIP1* expression can be induced by nitrate, the seedlings were grown for 7 days on medium containing 2.5 mM ammonium succinate as the sole N source, and then treated with 10 mM KNO_3_. We found that the expression of *FIP1* was not significantly altered in the roots following nitrate treatment (Supplementary Figure 1), indicating that *FIP1* expression is not induced by nitrate.

### *FIP1* Is Mainly Expressed in the Vascular Tissues of the Leaves and Roots

**FIGURE 3:**
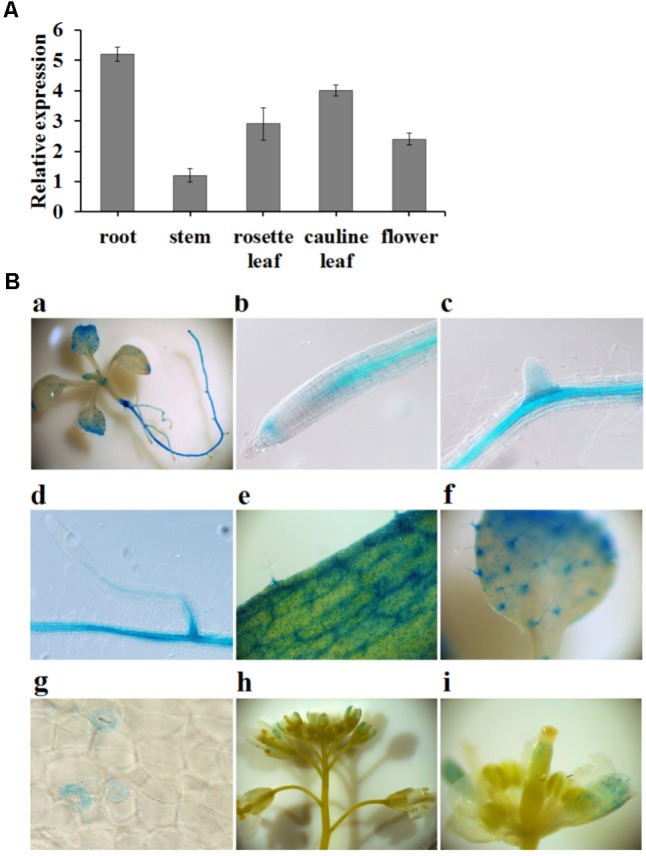
The expression pattern of *FIP1*. **(A)** Relative expression levels of *FIP1* in different tissues quantified using qPCR. Tissues were harvested either from 7-days-old plants (seedlings and roots) grown in 1/2 MS solution or 6-week-old plants (leaves, stems, and flowers) grown in soil. Error bars represent the SD of the biological replicates (*n* = 4). **(B)** Histochemical staining of GUS activity in transgenic plants expressing *Pro_FIP1_*::*GUS*. GUS activity was detectable in the (a) whole seedling, (b) root tip, (c,d) lateral roots, (e) rosette leaves, (f) cotyledons, (g) stomata, and (h,i) flowers.

To further explore the regulation of the nitrate response by *FIP1*, its expression pattern was investigated using qPCR. *FIP1* was found to be expressed in all tissues investigated, but was particularly highly expressed in the roots and leaves (**Figure 3A**). Histochemical analyses using transgenic lines harboring the *GUS* gene driven by the *FIP1* promoter revealed that *FIP1* was predominantly expressed in the vascular tissues of the roots and leaves (**Figure 3B**). GUS staining was also observed in the trichomes and stomata on the leaves, as well as in the flowers (**Figure 3B**). The expression profile of *FIP1* suggests that it may function in the nitrate signaling pathway of several tissues.

### *FIP1* Regulates the Uptake and Allocation of Nitrate Between the Shoot and Root

**FIGURE 4:**
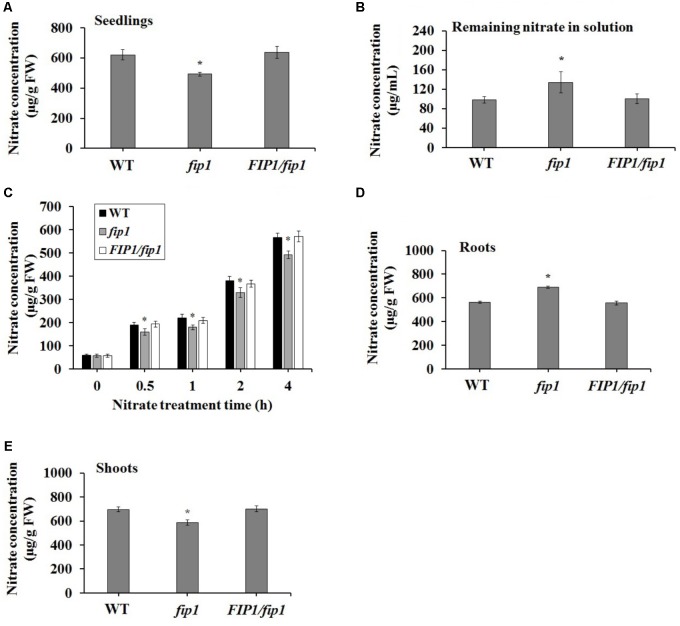
*FIP1* affects nitrate uptake and accumulation. Seedlings were grown in ½ MS solution for 7 days and their nitrate content was quantified. **(A)** The nitrate concentration in the seedlings. **(B)** The nitrate concentration remaining in the solution after 7 days of seedlings growth. **(C)** Nitrate uptake in seedlings grown for 7 days in 2.5 mM ammonium succinate and then treated with 5 mM KNO_3_ for the indicated periods. The nitrate concentration in the roots **(D)** and shoots **(E)**. Error bars represent the SD of the biological replicates (*n* = 5). Asterisks indicate significant differences in the transgenic lines in comparison with the WT (*p* < 0.05, *U*-test). FW, fresh weight.

Some nitrate regulators affect the accumulation of nitrate within the plant (Castaings et al., 2009; Wang et al., 2009; Xu et al., 2016; Li et al., 2017). To test the physiological effects of *FIP1*, we measured the nitrate content of plants grown on ½ MS medium. Nitrate accumulation was significantly decreased in the *fip1* seedlings compared with the WT (**Figure 4A**). This decreased nitrate content may result from defects in nitrate uptake and/or increased nitrate assimilation; therefore, we determined the remaining nitrate concentration in the solution after 7 days of seedling growth. The remaining nitrate concentration in the solution was significantly higher for *fip1* than for WT (**Figure 4B**). We tested the nitrate uptake of seedlings grown in the presence of 2.5 mM ammonium succinate for 7 days and then treated with 5 mM KNO_3_ for different durations, and found that the nitrate content was reduced significantly in the *fip1* mutant at the time points tested (**Figure 4C**). Taken together, these results indicate that *FIP1* affects nitrate uptake. To further investigate the distribution of nitrate in the plants, we quantified the accumulation of nitrate in both the shoots and roots. The nitrate content was higher in the roots but lower in the shoots of *fip1*, and this phenotype was recovered in the *FIP1*/*fip1* complementation line (**Figures 4D,E**), suggesting that the allocation of nitrate within the plant is altered in the *fip1* mutant. Taken together, these findings show that *FIP1* regulates nitrate uptake and allocation.

**FIGURE 5:**
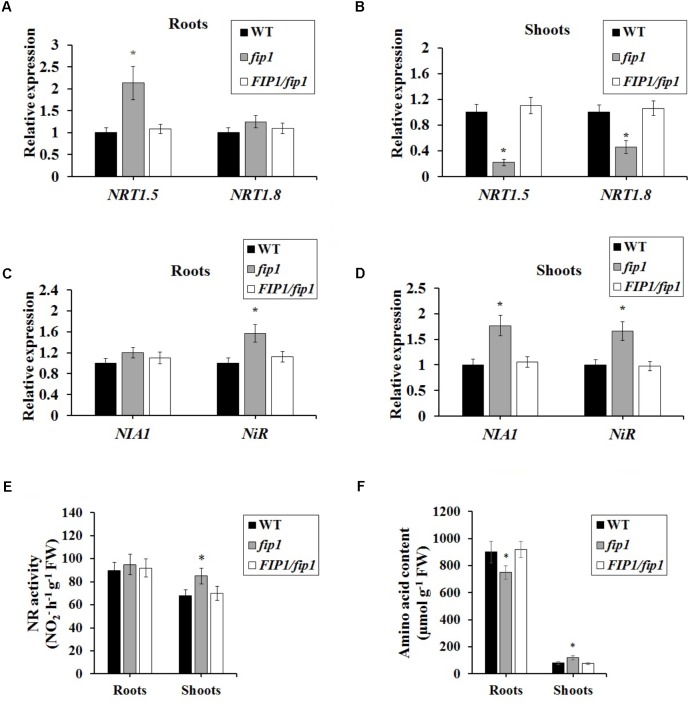
*FIP1* regulates the expression of genes involved in nitrate transport and assimilation in the roots and shoots of seedlings grown for 7 days in ½ MS solution. **(A,B)** The relative expression levels of nitrate transport genes *NRT1.5* and *NRT1.8* were determined in the roots **(A)** and shoots **(B)** using qPCR. **(B,C)** The expression levels of the nitrate assimilation genes *NIA1* and *NiR* were detected in the roots **(C)** and shoots **(D)** using qPCR. **(E)** Nitrate reductase (NR) activity and **(F)** amino acid content in *fip1* roots and shoots. Error bars represent the SD of the biological replicates (*n* = 5). Asterisks indicate significant differences in the transgenic lines in comparison with the WT (*p* < 0.05, *U*-test).

The decreased nitrate content in *fip1* led us to investigate the expression of genes known to be involved in nitrate transport and assimilation. For this assay, steady state levels of mRNA after a week of growth on nitrate were determined by growing seedlings on ½ MS for 7 days then harvesting roots and shoots separately for RNA extraction. Our qPCR analysis showed that *NRT1.5* expression was increased in the roots of *fip1* compared with the WT, while the expression of *NRT1.5* and *NRT1.8* was decreased in the *fip1* shoots in comparison with the WT (**Figures 5A,B**). These phenotypes were recovered in the *FIP1*/*fip1* complementation line. No significant differences in expression were found for the other genes tested (Supplementary Table 1). *NRT1.8* functions in nitrate unloading from the xylem to the shoots (Li et al., 2010). Therefore, the decreased expression of *NRT1.8* in the shoots may result in the lower nitrate content in the shoots but higher in the roots of *fip1*.

We also determined the expression levels of the nitrate assimilation-related genes. The expression levels of *NIA1* and *NiR* were much higher in *fip1* than in the WT (**Figure 5C**), while in the roots only the expression of *NiR* was higher in *fip1* (**Figure 5D** and Supplementary Table 2). Furthermore, we detected the NR activity and amino acid content of the plants. The NR activity in the shoots of *fip1* was higher than that of the WT (**Figure 5E**), while the amino acid content was lower in the roots and higher in the shoots of *fip1* compared with the WT (**Figure 5F**). These results demonstrate that *FIP1* also affects the expression of the genes involved in nitrate assimilation, which may be another reason for the lower nitrate content in the shoots of *fip1*.

### *FIP1* Regulates the Expression of *CIPK8* and *CIPK23* in the Presence of Nitrate and Functions in the Same Nitrate-Signaling Pathway as CPSF30

**FIGURE 6:**
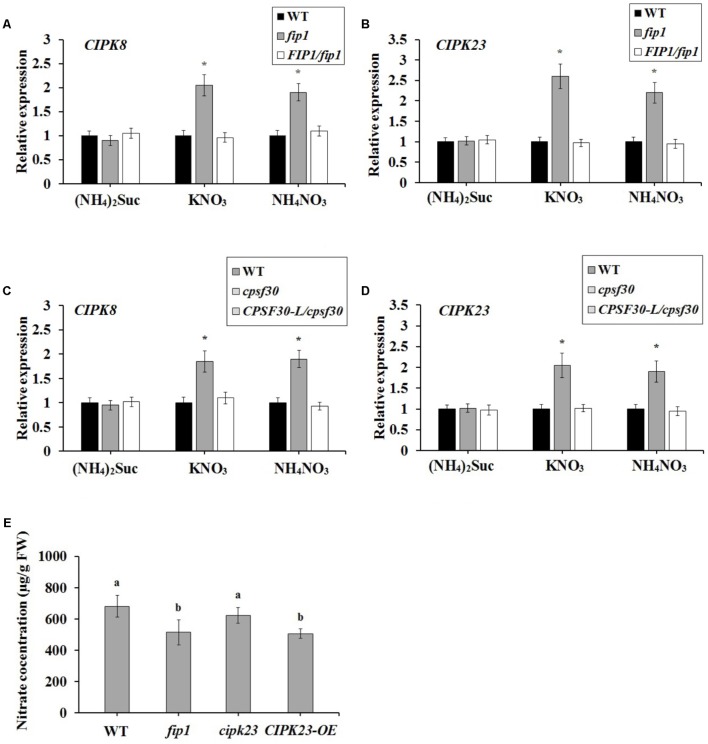
*FIP1* regulates the expression of *CIPK8* and *CIPK23* and affects seedling nitrate content in the presence of nitrate. RNA was extracted from seedlings grown for 7 days with various sources of nitrogen [(NH_4_)_2_Suc, KNO_3_, or NH_4_NO_3_ media]. The expression levels of *CIPK8* **(A)** and *CIPK23* **(B)** in the *fip1* mutant, determined using qPCR. The expression levels of *CIPK8* **(C)** and *CIPK23* **(D)** in the *cpsf30* mutant, determined using qPCR. Error bars represent the SD of the biological replicates (*n* = 4). **(E)** Nitrate content in the *CIPK23* overexpression line (*CIPK23-OE*) grown in ½ MS medium for 7 days. Error bars represent the SD of the biological replicates (*n* = 5). Asterisks and different letters indicate significant differences to the WT (*p* < 0.05, *U*-test).

To further explore the relationship between *FIP1* and the previously characterized nitrate regulatory genes, we grew nitrate regulation mutant plants, lacking *CPSF30*, *NRT1.1*, *NLP7*, *CIPK8*, *CIPK23*, or *NRG2*, for 7 days under three N sources (2.5 mM ammonium succinate, 10 mM KNO_3_, or 10 mM NH_4_NO_3_), and then quantified their expression of *FIP1*. There was no significant difference in the expression of *FIP1* between the WT and each mutant under the different N sources (Supplementary Figure 2), indicating that the above nitrate regulatory genes do not regulate its expression. We also detected the expression of these genes in *fip1*, revealing that the expression of *CIPK8* and *CIPK23* in *fip1* was significantly increased in comparison with the WT in the presence of nitrate, and that these changes were recovered in the *FIP1*/*fip1* complementation line (**Figures 6A,B**). No significant differences were found for the other genes tested (Supplementary Table 3). *CIPK8* and *CIPK23* are important regulatory genes involved in nitrate signaling (Ho et al., 2009; Hu et al., 2009); therefore, our results suggest that *FIP1* may play an essential role in the PNR by regulating the expression of the nitrate regulatory genes *CIPK8* and *CIPK23*.

The expression of *CIPK8* and *CIPK23* is also known to be controlled by *NRT1.1* (Ho et al., 2009; Hu et al., 2009). As CPSF30-L regulates the expression of *NRT1.1*, we investigated whether *CPSF30-L* affected the expression of *CIPK8* and *CIPK23.* We found that *CIPK8* and *CIPK23* expression was significantly higher in the *cpsf30* mutant than in the WT in the presence of nitrate, a phenotype that was recovered in the *CPSF30-L/cpsf30* complementation line (**Figures 6C,D**). These results demonstrate that, like *FIP1*, *CPSF30-L* can modulate the expression of *CIPK8* and *CIPK23.*

CIPK23 was previously reported to phosphorylate Thr101 of NRT1.1, thereby facilitating a shift to its high-affinity status (Ho et al., 2009). In theory, the increased expression of *CIPK23* may strengthen the phosphorylation of NRT1.1, enhancing its high-affinity nitrate transport activity and reducing nitrate uptake in the presence of sufficient nitrate. We therefore quantified the nitrate content of the *CIPK23* overexpression line (*CIPK23*-OE). The nitrate content of this line was reduced, whereas no significant difference was observed between the nitrate contents of *cipk23* and the WT (**Figure 6E**). These results suggest that the increased expression of *CIPK23* in *fip1* may enhance the phosphorylation of NRT1.1, resulting in the lower nitrate accumulation of this mutant.

**FIGURE 7:**
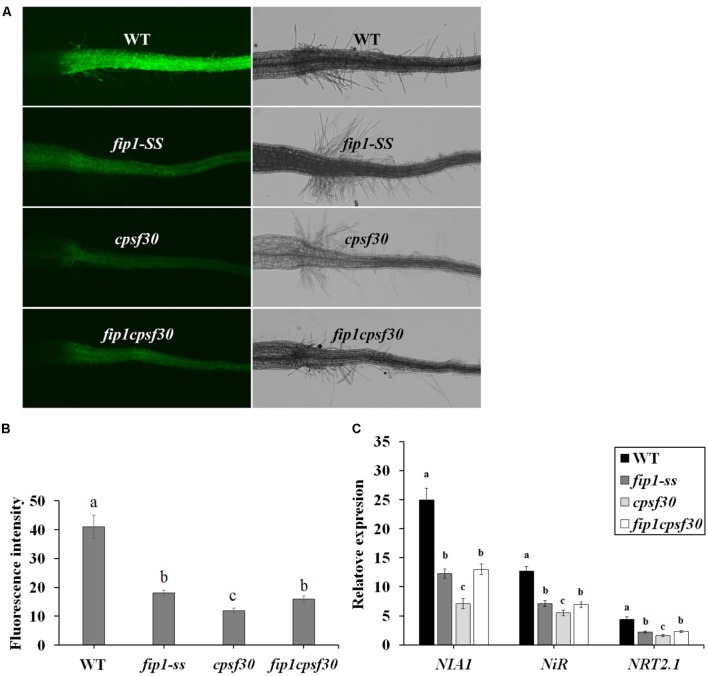
*FIP1* and *CPSF30* function in the same nitrate signaling pathway. **(A)** Nitrate responsiveness in 4-days-old WT, *fip1*, *cpsf30*, and *fip1cpsf30* seedlings grown in KNO_3_ medium, revealed using a fluorescent reporter system (NRP-YFP). **(B)** Quantification of root fluorescence in the plants described in **(A)**. Error bars represent the SD of the biological replicates (*n* = 60). **(C)** The expression of nitrate-responsive genes in the roots of WT, *fip1*, *cpsf30*, and *fip1cpsf30* plants. The seedlings were grown for 7 days on the medium containing 2.5 mM ammonium succinate as the sole nitrogen source, and then treated with 10 mM KNO_3_ or 10 mM KCl as a control for 2 h. The transcripts of the nitrate-responsive genes were quantified using qPCR. Error bars represent the SD of the biological replicates (*n* = 4). Asterisks and different letters indicate significant differences (*p* < 0.05, *U*-test).

To further investigate the relationship between *FIP1* and *CPSF30-L*, we constructed a double mutant (*fip1cpsf30*) by crossing *fip1-SS* and *cpsf30*. RT-PCR results showed that the expression of *FIP1* was undetectable and sequencing results exhibited that the point mutation of the *cpsf30* mutant was present in the double mutant (Supplementary Figure 3). The fluorescence intensity, indicating nitrate responsiveness, was significantly lower in *fip1* than in the WT but higher than in *cpsf30*, while no significant difference was found between *fip1* and the *fip1cpsf30* double mutant (**Figures 7A,B**), suggesting that *FIP1* and *CPSF30-L* may regulate the nitrate response via the same pathway. The expression levels of the nitrate-responsive genes *NIA1*, *NiR*, and *NRT2.1* were found to be higher in the *fip1cpsf30* double mutant than in *cpsf30*, while no significant difference in expression was observed between the *fip1cpsf30* double mutant and the *fip1* single mutant (**Figure 7C**). This further confirms that *FIP1* and *CPSF30-L* function in the same nitrate-signaling pathway. The similar phenotype of *fip1* mutant and the double mutant may be explained by the possibility that FIP1 and CPSF30-L may be part of a larger complex. Loss of FIP1 can results in a change in the complex that reduces the complex’s activity by a certain amount. Loss of CPSF30-L may result in a larger change that reduces activity more than loss of FIP1. Loss of both genes results in a change that mimics loss of FIP1 alone. This would happen if CPSF30-L binding requires FIP1 binding. Loss of FIP1 prevents CPSF30-L binding so that the phenotype of double mutant looks like that of the *fip1* single mutant.

**FIGURE 8:**
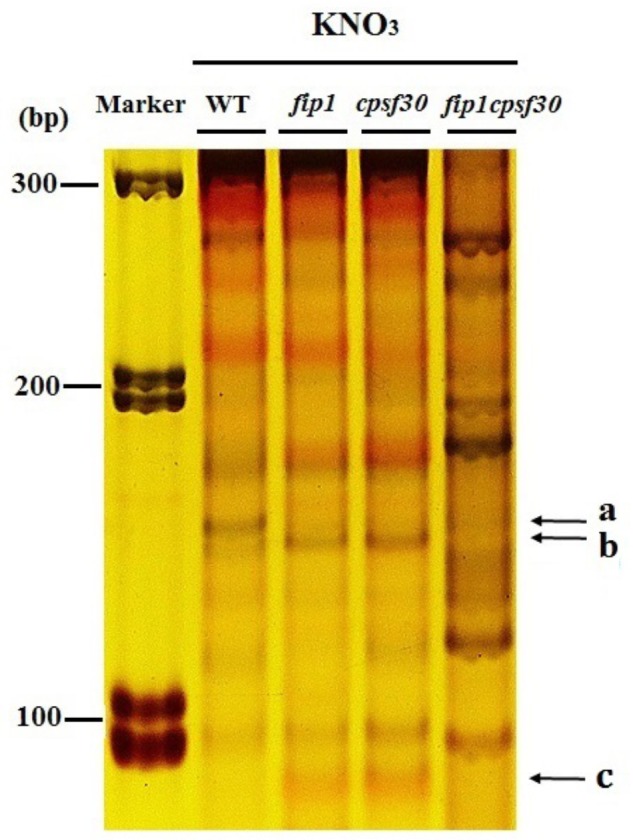
*FIP1* can affect the APA in 3′-UTR of *NRT1.1* mRNA. Polyacrylamide gel electrophoresis (PAGE) was run to separate the amplified bands of different 3′-UTR polyadenylated forms of *NRT1.1* in WT, *fip1*, *cpsf30*, and *fip1cpsf30*. The seedlings were grown on 10 mM KNO_3_ for 7 days and total RNA was extracted for RT-PCR.

Previous study has shown that *CPSF30-L* can affect APA of 3′-UTR in *NRT1.1* mRNA (Li et al., 2017). Since *FIP1* and *CPSF30* function in the same nitrate signaling pathway, it’s possible that *FIP1* also affects the APA of the *NRT1.1*. Therefore, 3′-UTR of *NRT1.1* was amplified by nested PCR from the WT, *fip1*, *cpsf30*, and *fip1cpsf30* seedlings grown on KNO_3_ medium. The results showed that there was a band (marked as a in **Figure 8**) in the WT, but this fragment was almost invisible in *fip1*, *cpsf30*, and *fip1cpsf30* double mutant while a lower band (marked as b) was more obvious in *fip1* and *cpsf30* mutants compared to that in WT (**Figure 8**). In addition, the *fip1* and *cpsf30* mutants contained a single band (marked as c) which was almost invisible in the WT. These results indicate that *FIP1* and *CPSF30-L* can similarly affect the APA of the 3′-UTR of *NRT1.1* mRNA. However, the bands b and c were almost invisible in *fip1cpsf30* double mutant, implying that the APA of 3′-UTR in *NRT1.1* mRNA in the double mutant may be more complicated.

## Discussion

In *Arabidopsis*, *CPSF30* has two spliced forms, a smaller one (*CPSF30-S*) and a larger one (*CPSF30-L*) (Delaney et al., 2006). *CPSF30-S* plays a role in a number of distinct developmental processes and physiological responses, partly resulting from a global shift in the poly(A) site choice of numerous responding genes (Zhang et al., 2008; Bruggeman et al., 2014; Liu et al., 2014); however, the function of *CPSF30-L* was largely unknown (Delaney et al., 2006). Recently, we reported that a nitrate regulatory mutant, Mut65, lacked *CPSF30* function (Li et al., 2017). Further investigation demonstrated that *CPSF30-L* and not *CPSF30-S* was responsible for the nitrate signaling (Li et al., 2017). It is known that CPSF30-S can interact with FIP1, a putative core component of a pre-mRNA processing complex (Addepalli and Hunt, 2007) and that this interaction regulates the nuclease activity of CPSF30. However, whether CPSF30-L could interact with FIP1 and whether *FIP1* was involved in nitrate signaling were not known. The data we present here indicate that the N-terminus of FIP1 does indeed interact with CPSF30-L (**Figure 1**) and plays an important role in nitrate signaling.

To determine whether *FIP1* participates in nitrate signaling, we quantified the expression of the nitrate-responsive genes in *fip1* and found that this mutant was defective in the PNR (**Figure 2**). Our histochemical assay and qPCR analysis showed that *FIP1* was mainly expressed in the stele of the primary root, basal lateral root, vascular tissue of the leaf, and stomata, and was not induced by nitrate (**Figure 3** and Supplementary Figure 1). The expression profile of *FIP1* was coincident with that of *CPSF30* (Li et al., 2017). The common expression sites of both genes further support the suggestion that FIP1 interacts with CPSF30-L.

*CPSF30-L* is known to regulate nitrate uptake and the expression of the nitrate transporter gene, *NRT1.1* (Li et al., 2017). Here, we demonstrated that the nitrate content was lower in the *fip1* mutant seedlings because of its defective nitrate uptake (**Figures 4A–C**); however, the expression levels of the known genes involved in nitrate uptake, such as *NRT1.1* and *NRT1.2*, were not affected in *fip1* (Supplementary Table 1). Furthermore, the expression of *CIPK23* was increased in *fip1* (**Figure 6B**). As CIPK23 can phosphorylate NRT1.1 to switch its activity to have a high affinity for nitrate, the increased expression of *CIPK23* may upregulate the phosphorylation and nitrate transport activity of NRT1.1, reducing nitrate uptake under conditions with sufficient nitrate. Indeed, the nitrate concentration in *CIPK23*-OE was decreased (**Figure 6E**), suggesting that the increased expression of *CIPK23* might result in the lower nitrate content observed in the *fip1* mutant.

In addition, *FIP1* affects the distribution of nitrate between the roots and shoots. The *fip1* mutant accumulated more nitrate than WT in the roots, but less in the shoots (**Figures 4D,E**), in contrast to the *cpsf30* mutant, which accumulates less nitrate than the WT in both its roots and shoots (Li et al., 2017). The lower nitrate content in the shoots and higher in the roots of the *fip1* mutant may be due to the following possibilities: (1) the lower expression of *NRT1.8* in the shoots may decrease nitrate unloading from the xylem to the shoot tissues (**Figure 5B**); (2) the increased expression of the nitrate assimilation genes may cause more nitrate to be reduced in the shoots (**Figure 5D**). The NR activity and amino acid contents in the *fip1* mutant were indeed higher than in the WT (**Figures 5E,F**). In the *cpsf30* mutant, the decreased expression of *NRT1.8* and the increased expression of the nitrate assimilation genes in the shoots also led to a lower nitrate content in the shoots (Li et al., 2017). These results indicate that *FIP1* plays an important role in regulating nitrate uptake, transport, and assimilation.

We investigated the relationships between *FIP1* and the nitrate regulatory genes, revealing that *FIP1* functions as a negative regulator to modulate the expression of *CIPK8* and *CIPK23* in the PNR, similar to the role of *CPSF30-L* (**Figure 6**). It has been reported that both *CIPK8* and *CIPK23* are involved in regulating the nitrate response (Ho et al., 2009; Hu et al., 2009); thus, our results suggest that *FIP1* may play an essential role in the PNR by regulating the expression of *CIPK8* and *CIPK23*. Moreover, our genetic and molecular findings suggest that *FIP1* and *CPSF30-L* work in the same nitrate-signaling pathway (**Figure 7**), *FIP1* also alter the 3′-UTR of *NRT1.1*, similar with *CPSF30-L* (**Figure 8**); however, the *fip1* and *cpsf30* mutants have some phenotypic differences, such as their nitrate distributions and their regulation of *NRT1.1* expression, reflecting the complexity of nitrate regulation.

*FIP1* is a core component of a pre-mRNA processing complex that is conserved between plants, yeast, and humans (Preker et al., 1995; Kaufmann et al., 2004; Forbes et al., 2006). In plants, FIP1 is an RNA-binding protein and its N-terminus (containing 137 amino acids) can interact with poly(A) polymerase and stimulate its activity. In addition, the N-terminus of FIP1 interacts with other protein machinery involved in the 3′-end processing of pre-mRNAs such as *CstF77*, *CFIm-25*, *PabN1*, and *CPSF30* (Forbes et al., 2006). Furthermore, it has been reported that three distinct hubs centered around *FIP1*, *CPSF100*, and *CLPS* are involved in poly(A) processing in *Arabidopsis* (Hunt et al., 2008). The biological function of FIP1 is unknown; however, the findings presented here indicate that *FIP1* plays an important role in nitrate signaling and the regulation of nitrate uptake, transport, and assimilation.

In *Arabidopsis*, the 28-kDa CPSF30-S subunit contains three zinc finger motifs and functions in polyadenylation, while the 65-kDa CPSF30-L subunit contains a YTH (YT521 homology) RNA-binding domain in addition to three zinc fingers (Delaney et al., 2006). In both splicing forms, the first zinc finger binds RNA and the third zinc finger has nuclease activity, but the function of the second zinc finger domain is unknown. FIP1 was previously shown to interact with the third zinc finger of CPSF30-S, thereby regulating its nuclease activity (Addepalli and Hunt, 2007). Furthermore, a disulfide bond can be formed between the side chains of two cysteine residues in the third zinc finger domain, which, when reduced, results in the loss of endonuclease activity in CPSF30-S (Addepalli et al., 2010). *CPSF30-S* plays a variety of roles in a number of distinct developmental and physiological responses, partly resulting from a global shift in the poly(A) site choice of the corresponding genes (Zhang et al., 2008; Bruggeman et al., 2014; Liu et al., 2014); however, whether *CPSF30-L* functions in the same way to influence APA remains unknown. Interestingly, a mutation in the third zinc finger motif of CPSF30-L affects the poly(A) processing of *NRT1.1* mRNA, resulting in the altered expression of *NRT1.1* (Li et al., 2017). These findings suggest that CPSF30-L mediates APA function in a similar manner to CPSF30-S; therefore, the function of CPSF30-L in regulating nitrate signaling may depend on its endonuclease activity. Here, we found that FIP1 could interact with CPSF30-L and also alter the APA in the 3′-UTR of *NRT1.1. FIP1* and *CPSF30-L* function in the same pathway to regulate nitrate signaling. There results suggest that *FIP1* and *CPSF30-L* may modulate the expression of nitrate related genes through affecting their APA process.

## Author Contributions

YW and NC designed the project. CW, WZ, ZhL, and YB performed the experiments. YW, CW, and ZeL analyzed the data. YW, NC, and CW wrote the manuscript.

## Conflict of Interest Statement

The authors declare that the research was conducted in the absence of any commercial or financial relationships that could be construed as a potential conflict of interest.

## Abbreviations

APA: alternative polyadenylation
CPSF: cleavage and polyadenylation specificity factor
GUS: β-glucuronidase
N: nitrogen
NiR: nitrite reductase
NR: nitrate reductase
NUE: nitrogen use efficiency
PNR: primary nitrate response
YFP: yellow fluorescent protein
