# Supplementary material for: FIP1 Plays an Important Role in Nitrate Signaling and Regulates CIPK8 and CIPK23 Expression in Arabidopsis
Source: Front Plant Sci. 2018 May 4;9:593. doi: 10.3389/fpls.2018.00593 (PMC5945890; doi:10.3389/fpls.2018.00593)
Supplement: Supplementary file 1 [file Data_Sheet_1.DOCX]

**Supplementary data**


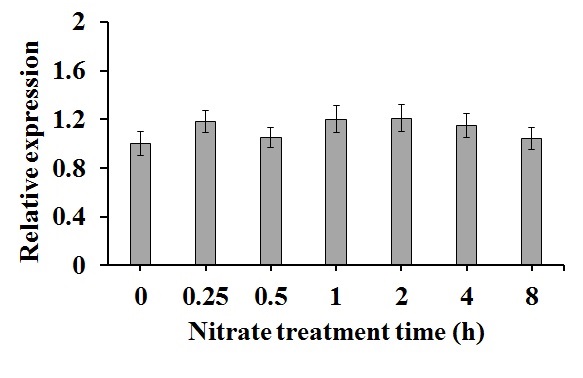


**Supplementary Figure 1 *FIP1* expression is not induced by nitrate treatment.**

The seedlings were grown for 7 d on a medium containing 2.5 mM ammonium succinate as the sole nitrogen source, and then treated with 10 mM KNO_3_ or 10 mM KCl as a control for the indicated periods. *FIP1* expression in the roots was determined using qPCR. Error bars represent the SD of the biological replicates (n=4).

**
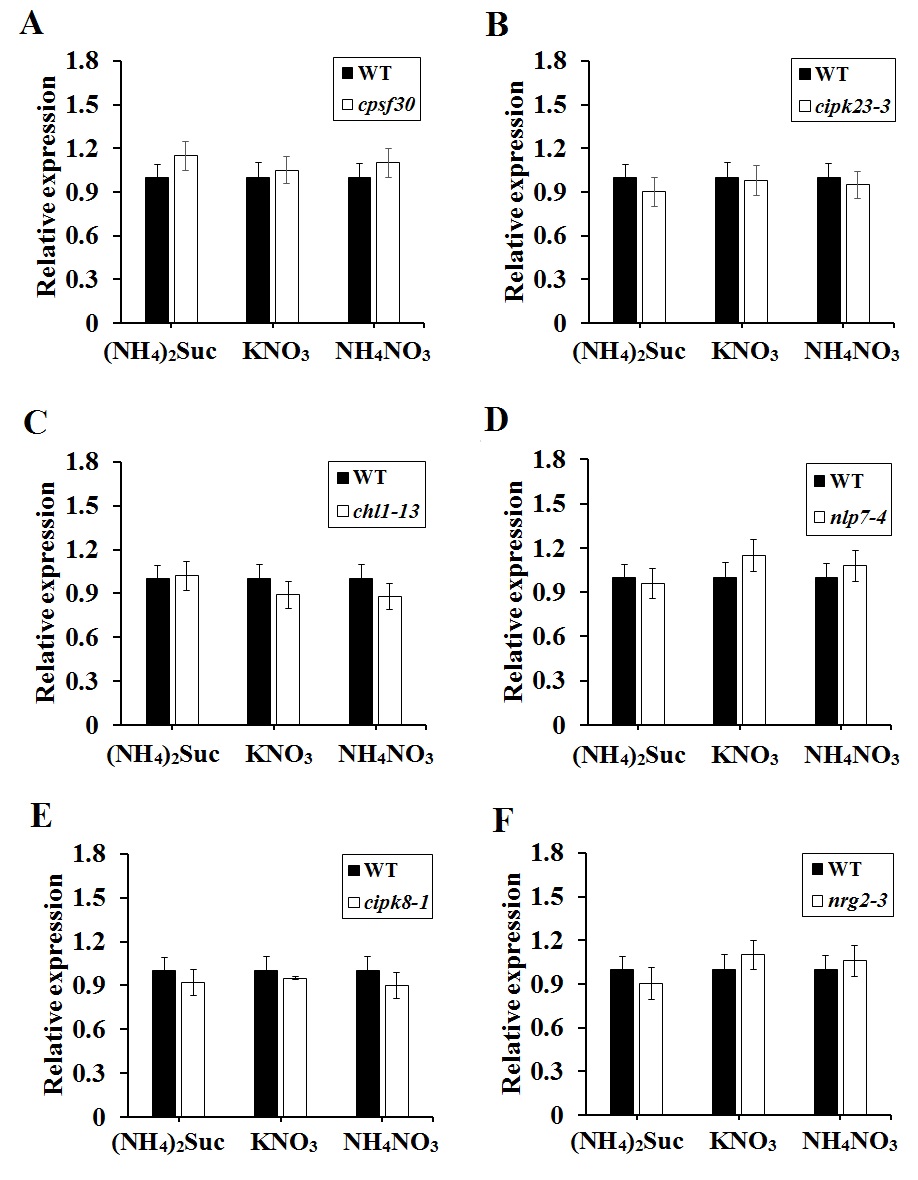
**

**Supplementary Figure 2 *FIP1* expression in various nitrate-regulatory gene mutants.**

The seedlings were grown for 7 d in media containing various nitrogen sources ((NH_4_)_2_Suc, KNO_3_, or NH_4_NO_3_). The expression levels of *FIP1* in *cpsf30* (**A**), *cipk23-3* (**B**), *chl1-13* (**C**), *nlp7-4* (**D**), *cipk8-1* (**E**), and *nrg2-3* (**F**) were detected using qPCR. Error bars represent the SD of the biological replicates (n=4).


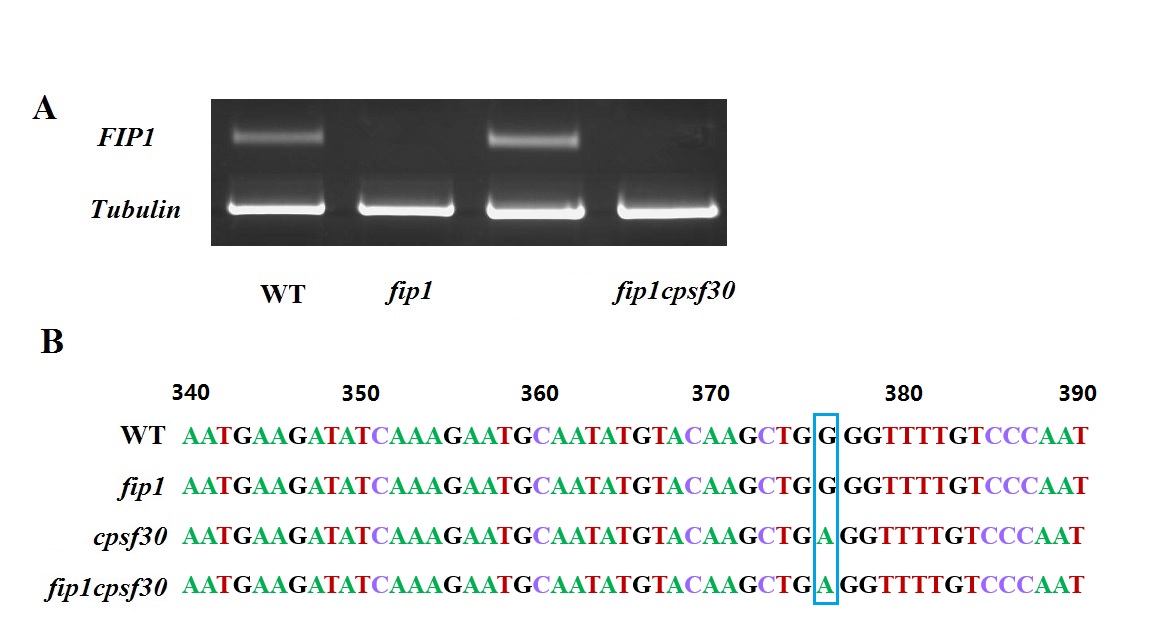


**Supplementary Figure 3 The detection of mutation in *fip1cpsf30* double mutant.**

(A) RT-PCR analysis of *FIP1* mRNA levels in WT, *fip1*, *cpsf30* and *fip1cpsf30* double mutant. Total RNA was isolated from 7-d-old seedlings grown on ½ MS. *TUB2* serves as the internal control. (B) Single nucleotide polymorphism (SNP) analysis by DNA sequencing in WT, *fip1*, *cpsf30* and *fip1cpsf30* double mutant. The *cpsf30* mutant was caused by EMS treatment with a point mutation in nucleotide 376 (G to A). To test this SNP in the *fip1cpsf30* double mutant using DNA sequencing.

**Supplementary Table 1 The expression of nitrate transport genes in the shoots and roots of *fip1***

| Shoots | Relative expression | Roots | Relative expression |
| --- | --- | --- | --- |
| *NRT1.1* | 1.38±0.24 | *NRT1.1* | 1.38±0.24 |
| *NRT1.2* | 1.42±0.07 | *NRT1.2* | 1.04±0.07 |
| *NRT1.4* | 1.43±0.18 | *NRT1.4* | 1.48±0.22 |
| *NRT1.7* | 1.53±0.39 | *NRT1.7* | 1.58±0.34 |
| *NRT1.9* | 1.53±0.36 | *NRT1.9* | 1.27±0.25 |
| *NRT1.11* | 1.27±0.15 | *NRT1.11* | 1.31±0.23 |
| *NRT1.12* | 1.37±.022 | *NRT1.12* | 0.74±0.22 |
| *NRT2.1* | 0.92±0.20 | *NRT2.1* | 0.85±0.21 |
| *NRT2.5* | 1.40±0.37 | *NRT2.2* | 0.71±0.22 |
| *NRT2.6* | 1.38±0.26 | *NRT2.5* | 0.81±0.15 |
| *NRT2.7* | 1.31±.0.25 | *NRT2.6* | 1.48±0.32 |
|  |  | *NRT2.7* | 1.11±0.15 |
|  |  |  |  |

The seedlings were grown for 7 d in ½ MS medium. The expression levels of known nitrate transport genes were determined using qPCR. n=4 biological replicates.

**Supplementary** **Table 2 The expression of nitrate assimilation genes in the shoots and roots of *fip1***

| Shoots | Relative expression | Roots | Relative expression |
| --- | --- | --- | --- |
| *NIA2*  *GLN1.1* | 0.85±0.02  0.91±0.11 | *NIA2*  *GLN1.1* | 0.95±0.13  0.91±0.17 |
| *GLN1.3* | 1.31±0.21 | *GLN1.3* | 0.96±0.14 |
| *GLN1.4* | 1.09±0.36 | *GLN1.4* | 1.14±0.21 |
| *GLN1.5* | 1.31±0.20 | *GLN1.5* | 1.28±0.17 |
|  |  |  |  |

The seedlings were grown for 7 d in ½ MS medium. The expression levels of known nitrate assimilation genes were determined using qPCR. n=4 biological replicates.

**Supplementary Table 3 The expression of nitrate regulatory genes in *fip1***

| **Gene** | **(NH_4_)_2_Suc** | **KNO_3_** | **NH_4_NO_3_** |
| --- | --- | --- | --- |
| *CPSF30* | 0.93±0.23 | 1.38±0.21 | 1.07±0.11 |
| *NRT1.1* | 1.14±0.32 | 1.08±0.20 | 1.16±0.28 |
| *NLP7* | 0.84±0.10 | 0.80±0.37 | 1.02±0.22 |
| *NRG2* | 1.06±0.09 | 0.99±0.27 | 0.95±0.16 |
| *LBD37* | 0.90±0.16 | *0.97*±0.18 | 1.02±0.21 |
| *LBD38* | 1.05±0.20 | *1.06*±0.21 | 1.02±0.22 |
| *LBD39* | 0.97±0.21 | *0.91*±0.18 | 0.94±0.18 |
| *IWS1* | 1.03±0.18 | *1.11*±0.11 | 1.09±0.15 |
| *TCP20* | 1.02±0.19 | *1.08*±0.15 | 1.10±0.14 |
| *TGA1* | 1.05±0.16 | *1.07*±0.21 | 1.02±0.22 |
| *TGA4* | 1.02±.020 | *0.96*±0.23 | 1.05±0.21 |
| *SPL9* | 1.05±0.18 | *1.08*±0.22 | 1.10±0.16 |
|  |  |  |  |

The seedlings were grown for 7 d in media containing various nitrogen sources ((NH_4_)_2_Suc, KNO_3_, or NH_4_NO_3_ media). The expression levels of some nitrate regulatory genes in *fip1* were determined using qPCR. n=4 biological replicates.
